# Pre-diabetes and diabetes are independently associated with adverse cognitive test results: a cross-sectional, population-based study

**DOI:** 10.1186/s12902-018-0318-3

**Published:** 2018-12-04

**Authors:** Elin Dybjer, Peter M. Nilsson, Gunnar Engström, Catherine Helmer, Katarina Nägga

**Affiliations:** 1 Department of Clinical Sciences, Lund University, Clinical Research Centre, Skane University Hospital, S-20502 Malmö, Sweden; 2 University of Bordeaux, Inserm, Bordeaux Population Health Research Center, team LEHA, UMR 1219, F-33000 Bordeaux, France; 3 Department of Acute Internal Medicine and Geriatrics, Linköping University, S-581 85 Linköping, Sweden; 4 Clinical Memory Research Unit, Department of Clinical Sciences Malmö, Lund University, S-205 02 Malmö, Sweden

**Keywords:** Ageing, Cognition, Diabetes, Epidemiology, Glucose, OGTT, Physical activity, Vascular

## Abstract

**Background:**

Diabetes is a risk factor for cognitive impairment, but whether there is also a link between pre-diabetes and cognitive dysfunction is not yet fully established. The aim of this observational study was to investigate associations between pre-diabetes/diabetes and cognitive test results, and also between glucose levels measured during the Oral Glucose Tolerance Test (OGTT) and cognitive outcomes.

**Methods:**

During 2007–2012, in all 2994 people (mean age 72 years), residing in Malmö, Sweden, underwent a clinical examination including the OGTT, cardiovascular measurements including carotid-femoral pulse wave velocity (c-f PWV) and two cognitive tests, the Mini Mental State Examination (MMSE), measuring global cognitive function, and A Quick Test of Cognitive Speed (AQT), measuring processing speed and executive functioning. Regression analyses were performed to investigate associations between: (a) categories of normal or impaired glucose metabolism, and (b) OGTT measurements, respectively, as exposure variables and cognitive test results as outcomes. Adjustments were made for demographics, lifestyle factors and cardiovascular risk factors.

**Results:**

Participants with pre-diabetes and diabetes scored slightly worse cognitive test results compared to the control group. Results of participants with a long disease duration of diabetes since the baseline examination 13 years earlier were poorer (mean AQT test time 17.8 s slower than controls, *p* < 0.001). Linear associations were found between fasting and 2-h glucose and cognitive outcomes in the whole population, but also in a sub-analysis including only individuals without diabetes (for 2-h glucose and MMSE results: B = − 2.961, *p* = 0.005). Associations were stronger for older or less physically active individuals. When adjusting for cardiovascular risk factors, most correlations were non-significant.

**Conclusions:**

Pre-diabetes and diabetes are associated with minor deficits in global cognitive function, processing speed and executive functioning. Long-standing diabetes is associated with bigger deficits. There appears to be a continuous inverse correlation between glucose levels and cognitive test results, also for people without diabetes. Associations are stronger in older and less physically active individuals. Cardiovascular factors are important mediating factors in the pathway between diabetes and cognitive dysfunction.

**Electronic supplementary material:**

The online version of this article (10.1186/s12902-018-0318-3) contains supplementary material, which is available to authorized users.

## Background

There is a growing body of evidence supporting that diabetes is a risk factor for cognitive impairment. Throughout the life course, mild cognitive decrements may develop as a consequence of long-term exposure to diabetes [1]. Type 2 diabetes also approximately doubles the risk of dementia [2]. The duration of diabetes has often been described as an important factor for these risk associations [3–5]. Neuroimaging studies have shown that diabetes-related cognitive impairment is characterized by similar pathological features as for vascular dementia, but also global brain atrophy [1]. Multiple cognitive domains are therefore often affected [6].

It is yet unclear whether pre-diabetes is a risk factor for cognitive decline, or not. Studies have been inconclusive, some showing that pre-diabetes is associated with worse cognitive performance [7, 8], particularly in domains such as processing speed [9], whereas others do not support these findings [10]. If this risk association becomes more firmly established, this could motivate interventions at earlier stages than today to prevent cognitive decline in people at risk of diabetes. Studies have so far mainly focused on interventions in manifest diabetes [11]. Furthermore, studies with refined methods of identifying early stages of impaired glucose metabolism, such as Oral Glucose Tolerance Testing (OGTT), are needed.

Markers of glycaemic control such as HbA_1c_, fasting glucose and 2-h post-OGTT glucose have previously been shown to be negatively correlated with cognitive test results, but studies have been inconsistent [12]. A reason may be that other cardiometabolic risk factors associated with diabetes have a more prominent effect on cognition than the actual glucose levels themselves. However, hyperglycaemia is associated with negative effects on nerve cells and on vascular tissue in mechanistic studies [13]. Some studies have shown that glucose levels correlate with cognitive test results also in individuals without diabetes [14–16], suggesting that there may be a continuous relationship between such markers and cognitive function also in the general population, but more studies are needed.

The mechanisms underlying cognitive decline and brain structural changes in people with diabetes are not well understood [17]. As diabetes is closely related to the metabolic syndrome, arterial stiffness [18, 19] and other cardiovascular risk factors, which also can act as risk factors for cognitive decline [20, 21], it is unclear to what extent these factors mediate the risk association.

In this study, we aimed to investigate associations between different stages of impaired glucose metabolism, but also glucose levels during the OGTT, and results of cognitive testing. We also investigated possible mediating and moderating effects of factors such as demographic, lifestyle-related and cardiovascular factors.

## Methods

### Participants

Data were obtained from the Malmö Diet and Cancer Study (MDCS), a population-based prospective cohort study from Malmö, Sweden. A baseline examination comprising 28,449 participants took place between 1991 and 1996, corresponding to a participation rate of approximately 40%. A cardiovascular cohort (MDCS-CV) was formed with 6103 of the participants who were randomly selected and re-invited to a follow-up examination during 2007 to 2012 when 3734 individuals participated, 76% of the surviving baseline participants. Enrolment details, reasons of loss to follow-up and information about potential health selection bias in the cohort have previously been described [22, 23]. The present study analyses cross-sectional data from the follow-up examination. People not born in Sweden, Norway or Denmark (*n* = 289), or with no data for country of birth (*n* = 113), were excluded as language fluency can affect cognitive test results. Participants missing essential cognitive or glucometabolic data were also excluded (*n* = 429). Some participants met both exclusion criteria (*n* = 91). The final study population comprised 2994 participants (740 excluded).

### Cognitive tests and sub-scores for specific domains

Participants were interviewed using two cognitive tests administered by trained research nurses during the follow-up examination: The Mini-Mental State Examination (MMSE) and A Quick Test of Cognitive Speed (AQT). For logistic reasons there was a time delay between physical examinations and subsequent cognitive testing sessions (mean 264 days). The MMSE is a global cognitive screening test in which orientation, memory, naming ability, ability to follow instructions, attention and copying of pentagons is tested [24]. It is widely used internationally and has been validated in Swedish populations [25]. The AQT test [26], a reliable screening test to detect early dementia [27], is a test of processing speed, attention and executive function. The test is timed, and involves naming first the colour, then the shape of 40 geometrical figures and then co-ordinating these activities. We also created scores for the following domains: *memory* (MMSE questions on orientation and delayed recall), *processing speed* (AQT part 1–2) and *executive functioning* (AQT part 3).

### Glucometabolic categories

At baseline, fasting glucose and HbA_1c_ were measured. At follow-up, fasting glucose was measured for all participants, and the OGTT (measurement of 2-h post-load glucose after administration of 75 g of glucose) was carried out in participants that did not have diabetes. Participants also reported in a questionnaire whether they had diabetes and whether they were medically treated for the disease both at baseline and at follow-up. Data on exact duration of diabetes was not available.

Participants were divided into groups of Normal Glucose Tolerance (NGT), pre-diabetes and diabetes. Pre-diabetes was defined as *impaired fasting glucose* (IFG) or *impaired glucose tolerance* (IGT). First, the categories were created using results of fasting and 2-h post-load glucose at follow-up, using the World Health Organization (WHO) 2006 criteria: *NGT* (fasting glucose < 6.1 mmol/l and 2-h glucose < 7.8 mmol/l), *IFG* (6.1 mmol/l ≤ fasting glucose < 7.0 mmol/l), *IGT* (7.8 mmol/L ≤ 2-h glucose < 11.1 mmol/L) and *screening-detected Type 2 diabetes* (fasting glucose ≥7.0 mmol/L or 2-h glucose ≥11.1 mmol/L). After that, participants that reported a diagnosis or drug treatment for diabetes at follow-up were added to the diabetes group, so as to re-classify those with treated diabetes and therefore normal glucose measurements into the diabetes category.

For the *post-hoc* analysis the diabetes category was then sub-divided into *long-term* diabetes, i.e. diabetes since the baseline examination 13 years earlier or before (fasting glucose ≥7 mmol/l, HbA_1c_ ≥ 52 mmol/l, treatment or self-reported diagnosis at baseline) and *short-term* diabetes, i.e. diabetes diagnosed after the baseline examination.

### Clinical examination

The follow-up examination took place at the Clinical Research Unit at Skåne University Hospital in Malmö, Sweden. Blood samples, a health questionnaire and clinical measures were administered. *Educational level* was categorized into ‘up to 10 years’, ‘11–12 years’ and ‘more than 12 years of schooling’; *physical activity level* into ‘sedentary spare time’, ‘moderate exercise’ and ‘regular exercise’; *smoking habits* into ‘never-smoker’, ‘former smoker’ or ‘current smoker’; and *alcohol consumption* into ‘no consumption’, ‘consumption below risk level’, and ‘consumption above risk level’ (> 9 standard alcohol units per week for women or > 14 for men according to Swedish guidelines). Finally, c-f PWV was measured with the SphygmoCor system, as described [19].

### Statistical analyses

Statistical analyses were performed using IBM SPSS version 24.0 for Mac OS X. A *p*-value less than 0.05 was considered significant. Missing data in covariates were imputed using multiple imputation with five consecutive imputations. Exposure and outcome variables were not imputed. The number of participants with any imputed data in the analyses was 187 in *Model 1* (6.2% of participants) and 493 (16%) in *Model 2* (see below). The variables with most imputed data were c-f PWV (*n* = 313), alcohol consumption (*n* = 119) and smoking habits (*n* = 73). In all other variables less than 12 cases were imputed.

Continuous variables were calculated into natural logarithmic values when needed to achieve normal distribution among residuals. To minimize ceiling effects that are usually present when analysing MMSE data in population-based studies, we used a normalizing transformation method that has been validated, creating a scale 0–100 instead of 0–30 as normally used in the test [28].

To compare differences in cognitive test results and covariates between glucometabolic categories, one-way between-groups analyses of variance (ANOVA) for continuous variables and Chi-square tests for categorical variables were carried out. Robust Test of Equality of means was used in cases when the homogeneity of variance assumption was violated (Levene’s test significant in ANOVA analyses).

Two adjustment models were used for the main analyses that follow. In *Model 1*, we adjusted for age, sex and education and also lifestyle factors physical activity level, smoking habits and alcohol consumption, as these factors could contribute to vascular cognitive impairment and therefore act as confounding factors. In *Model 2*, we additionally adjusted for cardiovascular risk factors: systolic blood pressure, heart rate, c-f PWV, waist circumference, total cholesterol, and anti-hypertensive, lipid-lowering and diabetes drug treatment. Although the relationship between diabetes and cognitive dysfunction is partly mediated by cardiovascular factors according to previous research, there are also non-vascular factors of importance [1]. We therefore made this model to see whether associations between diabetes and cognitive function are present independently of variations in cardiovascular factors. Some covariates were chosen over related variables based on their correlation with fasting glucose: i.e. systolic blood pressure over diastolic blood pressure and mean arterial pressure; waist circumference over body mass index (BMI) and waist/hip-ratio; and total cholesterol over other lipid variables.

Adjusted mean cognitive test results were compared between NGT (reference) to pre-diabetes and diabetes respectively in General linear model (GLM) analyses. Linear trends in cognitive test results across these three glucometabolic categories were also investigated in regression analyses. Post-hoc analyses were performed in the same way but with diabetes sub-divided into short-term and long-term diabetes.

As cognitive impairment caused by stroke can be regarded as a confounding factor, we excluded 174 participants with a history of stroke in a sensitivity analysis, repeating the analyses mentioned (data not shown). However, it could be argued that stroke is a disease that is in the pathway between diabetes and cognitive impairment, which is why we did not exclude these participants in the main analyses.

Multiple regression analyses were carried out to define whether interactions were present between covariates from the analyses (possible confounding factors) and the relationship between glucometabolic categories (as described above) and cognitive test results. When such interactions were significant, GLM analyses were performed to investigate the relationship between glucometabolic categories and cognitive function stratified for these variables.

Linear relationships between fasting and 2-h glucose respectively, and cognitive outcomes, were then investigated in multiple regression analyses. We also performed these analysis including only participants without diabetes.

To explore the data on fasting glucose longitudinally, as this was measured both during the baseline and follow-up examinations, we compared cognitive test results at follow-up between those that had IFG at baseline, at follow-up or both, with those who had NFG during both examinations (reference), in GLM analyses. Our hypothesis was that longer disease duration (i.e. IFG only at baseline or during both examinations) would be associated with worse cognitive outcomes because of organ damage accumulated over time.

Finally, we compared cognitive test results between the different classification methods for diabetes that were used during the baseline and follow-up examinations in further GLM analyses.

## Results

Characteristics of the study sample are presented in Table 1. The mean age was 72.4 years (range 61–85 years). Participants with pre-diabetes or diabetes were on average 1.1–1.2 years older and the proportion of men was higher in these categories than in the NGT category. Proportions of low physical activity and high, but also no alcohol consumption, were higher among participants with diabetes. There were no significant differences between the groups in educational level or smoking status. In general, cognitive test results were worse and cardiovascular risk factors more prevalent in the categories of pre-diabetes and diabetes compared to NGT. Total cholesterol levels were lower in the participants with diabetes, due to concomitant lipid-lowering treatment.

**Table 1:** Characteristics of the MDC CV Re-exam Cohort and Participants classified as NGT, Pre-diabetes and Diabetes

	Total study sample (*n* = 2994)	NGT (*n* = 1539)	Pre-diabetes (*n* = 902)	Diabetes (*n* = 553)	*P*-value (df = 3)
Covariates					
Age (years)	72.4 (5.58)	71.8 (5.56)	73.0 (5.70)	72.9 (5.31)	**< 0.001**
Sex (men/women, %)	40.2/59.8	35.7/64.3	43.3/56.7	47.9/52.1	**< 0.001**
Educational level (%)					
Low (≤ 10 years)	68.6	66.5	71.4	70.2	0.089
Medium (11–12 years)	9.46	9.62	9.20	9.40	
High (> 12 years)	22.2	23.8	19.4	20.4	
Physical activity (%)					
Sedentary spare time	7.08	5.20	7.76	11.2	**< 0.001**
Moderate exercise	75.7	74.3	77.9	76.0	
Regular exercise	17.2	20.5	14.3	12.7	
Smoking status (%)					
Never-smokers	45.3	46.8	44.4	42.8	0.105
Past smokers	45.0	44.6	43.9	48.0	
Present smokers	9.65	8.64	11.6	9.19	
Alcohol consumption (%)					
No consumption	23.1	20.3	24.6	28.2	**< 0.001**
Moderate consumption	64.4	67.8	62.1	58.6	
High consumption	12.5	11.9	13.3	13.2	
Systolic BP (mmHg)	143.2 (18.9)	141.4 (18.9)	143.8 (18.9)	147.4 (18.3)	**< 0.001**
Waist circumference (cm)	92.2 (12.5)	89.2 (11.5)	93.5 (12.4)	98.4 (11.5)	**< 0.001**
Heart rate (beats/min)	67.4 (11.1)	66.5 (10.4)	68.0 (11.4)	69.2 (12.0)	**< 0.001**
Pulse wave velocity (m/s)	10.5 (2.66)	10.2 (2.72)	10.7 (2.92)	11.3 (3.15)	**< 0.001**
Total cholesterol (mmol/l)	5.21 (1.07)	5.49 (1.01)	5.06 (1.03)	4.64 (1.05)	**< 0.001**
Fasting glucose (mmol/l)	6.09 (1.28)	5.46 (0.443)	6.17 (0.523)	7.77 (2.01)	**< 0.001**
2-h glucose (mmol/l)^a^	7.11 (1.54)	5.82 (1.16)	8.01 (1.65)	11.7 (0.02)	**< 0.001**
MMSE full test (/30p)	28.3 (1.81)	28.3 (1.65)	28.0 (1.83)	27.9 (2.00)	**<.0001**
AQT full test (time, s)	135.8 (32.3)	132.1 (28.3)	138.3 (33.7)	142.0 (38.6)	**< 0.001**
MMSE_memory score_ (/13p)	12.1 (1.02)	12.2 (0.957)	12.0 (1.05)	12.0 (1.11)	**< 0.001**
AQT_speed (Part 1–2)_ (time, s)	62.8 (14.5)	61.1 (12.9)	64.0 (14.8)	65.7 (17.2)	**< 0.001**
AQT_executive (Part 3)_(time, s)	73.0 (19.7)	71.0 (17.3)	74.4 (20.7)	76.3 (23.4)	**< 0.001**

Significant *p*-values are highlighted in **bold** text

^a^ For 2-h glucose (for which missing data was not imputed) *n* = 2671 for the total cohort, *n* = 1523 for NGT, *n* = 895 for pre-diabetes and *n* = 253 for diabetes

In Table 2, adjusted mean cognitive test results in categories of NGT, pre-diabetes and diabetes respectively are presented. *P*-values representing differences between the NGT category (reference) and pre-diabetes and diabetes respectively, as well as *p*-values for trends in cognitive test results across the three categories are also shown. There were very small differences in cognitive test results between the groups. The group with pre-diabetes had a mean difference in MMSE points (p) of 1.9/100 compared to NGT (on the normalized MMSE scale), and for the group with diabetes the difference was 2.0/100 (*p* < 0.01). Those with pre-diabetes were on average 3.0 s slower than the NGT group at the AQT test (mean test time for the whole cohort 135.8 s unadjusted), and the diabetes group 5.2 s slower than NGT. Differences in scores of cognitive sub-domains between NGT and pre-diabetes and diabetes, respectively, were also significant but very small (around 0.1p/12p difference for memory, and 1–3 s difference for processing speed and executive functioning). There were significant linear trends in cognitive test results across the categories, with negative trends for MMSE and the domain memory (measured in points) and positive trends for AQT and the domains processing speed and executive functioning (measured in time). When additionally adjusting for cardiovascular factors in *Model 2*, differences in cognitive test results between the categories and trends across the categories were in general non-significant.

**Table 2:** General linear models of adjusted mean cognitive test results of groups of participants with NGT, pre-diabetes and diabetes

	Model 1, n = 2994	Model 2, n = 2994
A. MMSE (points/100, normalized)		
NGT	80.4 (79.7–81.1)	80.0 (79.3–80.8)
Pre-diabetes	78.5 (77.6–79.4)**	78.3 (77.4–79.2)**
Diabetes	78.4 (77.2–79.5)**	79.8 (78.3–81.2)
P for trend across categories	**0.001**	0.143
B. AQT (time in seconds)		
NGT	130.8 (129.5–132.2)	131.8 (130.3–133.1)
Pre-diabetes	133.8 (132.0–135.5)*	134.0 (132.2–135.8)
Diabetes	136.0 (133.8–138.2)*	133.2 (130.5–135.9)
P for trend across categories	**< 0.001**	0.125
C. Memory (MMSE question 1 + 4, points/13)		
NGT	12.1 (12.1–12.2)	12.1 (12.0–12.2)
Pre-diabetes	12.0 (11.9–12.1)*	12.0 (11.9–12.1)*
Diabetes	12.0 (11.9–12.1)*	12.0 (11.9–12.2)
P for trend across categories	**0.009**	0.104
D. Processing speed (AQT part 1–2, time in seconds)		
NGT	60.6 (60.0–61.2)	61.0 (60.4–61.6)
Pre-diabetes	61.9 (61.2–62.7)*	62.1 (61.3–62.9)*
Diabetes	63.1 (62.1–64.1)***	61.6 (60.3–62.8)
P for trend across categories	**< 0.001**	0.129
E. Executive functioning (AQT part 3, time in seconds)		
NGT	69.9 (69.1–70.7)	70.3 (69.5–71.2)
Pre-diabetes	71.4 (70.4–72.5)	71.5 (70.1–72.6)
Diabetes	72.5 (71.2–73.9)***	71.2 (69.5–72.9)
P for trend across categories	**0.001**	0.178

* *p* < 0.05, **p < 0.01, ***p < 0.001 of difference in mean test results between NGT and each other category

Values are re-calculated from logarithmic values, apart from results of the MMSE, in which a normalization transformation method was used. All values are expressed as adjusted means (95% CI). For each cognitive outcome measurement, *p*-values for linear trends across categories are presented. Significant *p*-values for trend are highlighted in **bold** text

*Model 1:* Adjusted for age, sex, education, physical activity level, smoking habits and alcohol consumption

*Model 2:* Adjusted for factors in *Model 1* and cardiovascular factors: Systolic blood pressure, heart rate, c-f PWV, waist circumference, total cholesterol levels and medications (anti-hypertensive, anti-diabetic and lipid-lowering treatment)

In Additional file 1: Table S1, *post-hoc* analyses in which the diabetes category is divided into short-term and long-term diabetes, are presented. In these analyses, the differences between results of participants with NGT and long-term diabetes were greater. The difference in MMSE total score was 5.7 points out of 100 (*p* < 0.01), and the difference in AQT test time was 17.8 s (*p* < 0.001). When adjusting for cardiovascular factors in *Model 2* there were also significant differences in cognitive test results between participants with NGT and long-term diabetes apart from when analysing the MMSE total score. Trends of cognitive test results across the categories were also significant in this model, apart from when analysing the MMSE total score. When excluding participants with a history of stroke in an equivalent sensitivity analysis, results were essentially unchanged (data not shown).

In Table 3, correlations between continuous glucose measurements (fasting and 2-h) and MMSE and AQT results are presented. All correlations were significant in *Model 1* (adjusted for demographics and lifestyle factors), with negative associations for MMSE results (measured in points) and positive associations for AQT results (measured in test time). This was true both when including the whole study population (MMSE and 2-h glucose: B = − 2.147, *p* = 0.012; AQT and 2-h glucose: B = 0.033, *p* = 0.006) and also when only including participants without diabetes in a sub-analysis (MMSE and 2-h glucose: B = − 2.961, *p* = 0.005; AQT and 2-h glucose: B = 0.042, *p* = 0.004).

**Table 3:** Multiple linear regression analyses of linear relationships between *fasting* and *2 h-glucose* respectively and cognitive test results

	Model 1		Model 2	
	B	*p*	B	*p*
All participants				
Fasting glucose (*n* = 2991)				
MMSE total score	−5.325	**< 0.001**	−2.720	0.135
AQT total score	0.087	**< 0.001**	0.034	0.188
2 h-glucose (n = 2671)				
MMSE total score	−2.147	**0.012**	−1.787	**0.046**
AQT total score	0.033	**0.006**	0.023	0.072
All without diabetes				
Fasting glucose (*n* = 2484)				
MMSE total score	−8.323	**0.001**	−5.860	**0.030**
AQT total score	0.098	**0.004**	0.035	0.360
2 h-glucose (*n* = 2433)				
MMSE total score	−2.961	**0.005**	−2.563	**0.019**
AQT total score	0.042	**0.004**	0.030	**0.046**

*B* unstandardized regression coefficient. Significant *p*-values are highlighted in **bold** text

*Model 1:* Adjusted for age, sex, education, physical activity level, smoking habits and alcohol consumption

*Model 2:* Adjusted for factors in *Model 1* and cardiovascular factors: Systolic blood pressure, heart rate, c-f PWV, waist circumference, total cholesterol levels and medications (anti-hypertensive, anti-diabetic and lipid-lowering treatment)

In *Model 2* (additionally adjusted for cardiovascular factors), correlations were only significant between MMSE and 2-h glucose for the whole study population. In the sub-analysis with people without diabetes, all correlations were significant in this model apart from the relationship between fasting glucose and AQT. Correlations between glucose measurements and cognitive domains (memory, processing speed, executive functioning) were also significant in *Model 1*, but not consistently in *Model 2* (Additional file 1: Table S2).

The explained variance of the association between categories and markers of glycaemia on cognitive test results (presented in Tables 2 and 3) was low, in general less than 1%.

In Table 4, mean cognitive test results are compared between participants that had NFG both at baseline and at follow-up, and groups with IFG during one or both examinations. Having NFG both at baseline and at follow-up was associated with the best mean cognitive test results. Results were marginally poorer for those with IFG only at follow-up (newly diagnosed diabetes). Having IFG at baseline but not at follow-up (treated long-term diabetes) and having IFG during both examinations (dysregulated long-term diabetes) was associated with the poorest results. The mean AQT test time for both these groups of participants was 158.4 s compared to 135.2 for those with NFG during both examinations, *p* < 0.001.

**Table 4:** General linear models of adjusted mean cognitive test results across groups of participants with NFG or IFG at baseline and/or at follow-up. Adjusted for age, sex, education, physical activity level, smoking habits and alcohol consumption

	N	Mean cognitive test result (95% CI)	P for significant difference compared to NGT
A. MMSE (points/100, normalized)			
NFG at baseline and follow-up	2483	79.5 (78.9–80.0)	
IFG only at follow-up	320	79.4 (77.9–81.0)	0.955
IFG only at baseline	18	72.9 (66.5–79.3)	**0.034**
IFG at baseline and follow-up	43	74.9 (70.7–79.0)	**0.041**
B. AQT (time in seconds)			
NFG at baseline and follow-up	2483	135.2 (134.0–136.4)	
IFG only at follow-up	320	139.4 (136.0–142.8)	**0.026**
IFG only at baseline	18	158.4 (144.2–172.6)	**< 0.001**
IFG at baseline and follow-up	43	158.4 (149.2–167.7)	**< 0.001**

Significant *p*-values are highlighted in **bold** text

In Additional file 1: Table S3, different classification methods of diabetes are compared as regards cognitive test results. The results indicate that having diabetes is associated with worse mean cognitive test results compared to not having diabetes, irrespective of which classification method was used, although these differences were not always significant. There was one exception, i.e. there was no difference in mean results of MMSE in those with diabetes or without when classified by OGTT, which was not measured in participants with known diabetes, i.e. those classified as having diabetes had newly diagnosed diabetes.

We explored interactions between possible confounding factors (that were also covariates in the analyses) and glucometabolic categories in relation to cognitive function. Significant interactions were found for the variables age and physical activity regarding AQT results (data not shown), whereas no significant interactions were found for the variables sex, educational level, smoking habits, alcohol consumption, blood pressure, waist circumference, total cholesterol levels or c-f PWV. Associations between glucometabolic categories and cognitive outcomes stratified for age and physical activity are presented in Additional file 1: Table S4. The results indicate that the relationship between diabetes and poor AQT results is stronger in individuals that are older or exercise less.

## Discussion

In this cross-sectional population-based study, having pre-diabetes or diabetes was associated with minor deficits in global cognitive function, processing speed and executive functioning compared to having normal glucose tolerance (NGT). However, the differences were small and hardly considered as clinically relevant. In a sub-analysis it became apparent that differences in cognitive test results were greater between those with NGT and those with *long-term* diabetes (diagnosis since baseline 13 years earlier or before). Having IFG at baseline was also associated with worse cognitive test results than having IFG at follow-up. These findings taken together support the fact that early stages of impaired glucose metabolism are associated with mild cognitive decrements [1] but long-standing diabetes with more significant cognitive impairment, as well as the fact that diabetes duration is a predictor of cognitive outcomes [4].

Having pre-diabetes or diabetes was associated with minor differences in performance compared to NGT regarding the cognitive domains memory, processing speed and executive functioning. Having *long-term* diabetes was associated with poorer performance in these domains compared to controls, apart from memory performance in which there were only slight differences compared to results of the NGT category. These results are in line with other studies [6].

Fasting and 2-h glucose were both linearly associated with cognitive test results, both in the whole study population and in a sub-analysis including only participants without diabetes. This supports previous findings [14–16] and may imply that there are adverse effects of impaired glucose metabolism on cognition even at sub-clinical stages. However, longitudinal and interventional studies are needed to confirm these findings.

When adjusting our main two sets of analyses (glucometabolic categories and glucose levels, respectively, as exposure variables and cognitive test results as outcome variables) for cardiovascular risk factors in *Model 2*, we found that correlations were non-significant in most cases. This implies that cardiovascular factors largely mediate the relationship between impaired glucose metabolism and cognitive function in this elderly study population. This supports other findings that have shown that vascular damage is a key underlying process in diabetes-related cognitive impairment [1]. It is likely that both atherosclerosis and arteriosclerosis (arterial stiffness), impacting on the cerebral microcirculation, are important components of vascular damage in this context. It is well-known that diabetes is a risk factor for atherosclerosis-related co-morbidities such as stroke and myocardial infarction. In a sensitivity analysis in which we excluded participants with stroke, results were essentially unchanged, why it is likely that effects of stroke-related cognitive impairment were not great in this study.

Not many studies have adjusted for markers of arterial stiffness, such as PWV. Studies have shown that increased arterial stiffness in combination with hypotension may impair cognitive function in the elderly [29]. As pointed out by Scuteri, there may exist a so called Systemic Hemodynamic Atherosclerotic Syndrome (SHATS), involving a multitude of target organ damage related to vascular changes [30]. Furthermore, in the present Malmö cohort, correlations have been identified both between glucose levels and arterial stiffness [19] and between arterial stiffness and cognitive performance [21]. It is also likely that microvascular dysfunction precedes and contributes to the pathophysiological processes behind diabetes-related cognitive impairment [31].

Some correlations were still significant after cardiovascular adjustment, such as the sub-analysis of glucose levels (as exposure) and cognitive test results (as outcome) including only participants without diabetes. This may indicate that the mediating effects of cardiovascular factors are not as strong in individuals that have not yet developed diabetes. Other possible mechanistic factors behind the relationship between impaired glucose metabolism and cognitive dysfunction include oxidative stress in nerve cells and vascular tissue induced by hyperglycaemia [13], as well as accumulation of advanced glycation end products (AGEs), that may interact with the pathophysiological processes behind Alzheimer’s disease [32].

The relationship between diabetes and AQT results was stronger in participants that were older and/or exercised less. Cognitive reserve capacity generally falters with age. It is therefore likely that ageing entails vulnerability to adverse effects of diabetes on cognition. Physical activity is associated with positive effects on glucose metabolism and cognition separately, but no study to our knowledge has yet shown that it may modify the relationship between diabetes and cognitive function. Benefits of physical activity include improved cardiovascular and respiratory function, brain oxygen transport capability and mental health status [33]. Negative effects of physical inactivity on diabetes-related cognitive outcomes may act via some of these factors. Another factor that could be related to this association is presence of the Apo-lipoprotein E-ε4 (*APOE-ε4*) allele. Studies have shown that carriers may be more vulnerable to the insults of poor glycaemic control on cognition [34] but also less susceptible to positive effects on cognition that are related to physical activity [35].

The strengths of this study include the large sample size, the population-based setting, inclusion of OGTT as a diagnostic method, and the possibility to adjust for extensive cardiovascular risk factors including c-f PWV (arterial stiffness). The limitations include the cross-sectional study design, the time latency between physical and cognitive examinations and the inability to adjust for certain confounding factors such as depression, dietary intake and the *APOE-ε4* genotype, which taken together may greatly influence the risk of diabetes-associated cognitive impairment. Furthermore, we did not have the possibility to perform more sensitive neuropsychological cognitive tests on such a large number of participants, and therefore we could not study other cognitive domains. Finally, we did not involve markers of inflammation that could play an important role related to impaired glucose metabolism [36] and endothelial dysfunction, the latter a condition present already in normoglycaemic offspring to parents with diabetes [37].

When considering the generalizability of this study, it must be taken into account that this is an elderly Swedish urban population with a medium educational level. It is also likely that effects of the positive health selection bias are considerable. For example, the proportion of individuals with diabetes at baseline was 6.2% among those who attended the examination and 13.3% among those who did not. In addition, the non-attendees also had a generally worse cardiovascular risk factor profile [23].

The explained variance of the associations between diabetes or glucose levels and cognitive test results was small in this study, in general less than 1%, in contrast to results of a meta-analysis in which the explained variance of HbA_1c_ on cognition was calculated to be around 10% [12]. However, the present study is population-based and most cognitive tests and glucose values were normal, which may partly explain this difference. Furthermore, the MMSE is not a very sensitive test and may not cover the cognitive domains that are most often affected by diabetes.

We found a relationship between pre-diabetes and slight negative effects on cognition. It would be of interest to confirm this finding using a longitudinal approach in the same setting. Our findings also suggest that physical activity level is an important modifying factor in this context. More studies are needed to evaluate whether lifestyle or drug treatment interventions could prevent cognitive decline. Some have already been carried out but the results vary and the focus is on preventing manifest diabetes. For example, in the population-based Finnish Diabetes Prevention Study, frequent physical activity was associated with better cognitive performance [38]. In a prospective study, no differences were observed between groups with intensive or standard drug treatment for diabetes as regards cognitive test results or later dementia incidence [11]. There is, however, an ongoing study to determine whether randomized treatment with Linagliptin can improve cognitive outcomes over time [39].

## Conclusions

This large population-based study adds to the body of evidence that (a) long diabetes duration is associated with cognitive impairment that affects several cognitive domains; that (b) pre-diabetes and newly diagnosed diabetes are associated with mild cognitive decrements; and that (c) blood glucose levels, even within the upper end of the normal range, may be associated with slight negative effects on cognition. It also highlights the possible impact of cardiovascular factors as mediators for the associations, as well as the modifying effects of ageing and physical activity.

## Additional file

Additional file 1: **Table S1.** General linear models of adjusted mean cognitive test results across glucometabolic categories, a post-hoc analysis including diabetes with short and long duration. **Table S2.** Multiple linear regression analyses of linear relationships between fasting and 2 h-glucose, respectively, and cognitive sub-domains. **Table S3.** General linear models of adjusted mean cognitive test results for participants with and without diabetes, using different classification methods for diabetes. Adjusted for age, sex, education, physical activity level, smoking habits and alcohol consumption. **Table S4.** General linear models of adjusted mean AQT results for each glucometabolic category stratified for age and physical activity, adjusted for age, sex, education, physical activity level, smoking habits and alcohol consumption. (DOCX 36 kb)

## Abbreviations

APOE-ε4: Apo-lipoprotein E- ε4
AQT: A quick test of cognitive speed
C-f PWV: Carotid-femoral pulse wave velocity
GLM: General linear model
IFG: Impaired fasting glucose
IGT: Impaired glucose tolerance
MMSE: Mini mental state examination
NFG: Normal fasting glucose
NGT: Normal glucose tolerance
OGTT: Oral glucose tolerance test
