# Supplementary material for: Pre-diabetes and diabetes are independently associated with adverse cognitive test results: a cross-sectional, population-based study
Source: BMC Endocr Disord. 2018 Dec 4;18:91. doi: 10.1186/s12902-018-0318-3 (PMC6278035; doi:10.1186/s12902-018-0318-3)
Supplement: Supplementary file 1 — Table S1. General linear models of adjusted mean cognitive test results across glucometabolic categories, a post-hoc analysis including diabetes with short and long duration. Table S2. Multiple linear regression analyses of linear relationships between fasting and 2 h-glucose, respectively, and cognitive sub-domains. Table S3. General linear models of adjusted mean cognitive test results for participants with and without diabetes, using different classification methods for diabetes. Adjusted for age, sex, education, physical activity level, smoking habits and alcohol consumption. Table S4. General linear models of adjusted mean AQT results for each glucometabolic category stratified for age and physical activity, adjusted for age, sex, education, physical activity level, smoking habits and alcohol consumption. (DOCX 36 kb) [file 12902_2018_318_MOESM1_ESM.docx]

Revised version: **BEND-D-18-00314**

**MANUSCRIPT SUPPLEMENT (Tables S1-S4)**

**Pre-diabetes and diabetes are independently associated with adverse cognitive test results in a Swedish population-based cohort**

**Elin Dybjer** (corresponding author)

Department of Clinical Sciences, Lund University, Clinical Research Centre,

Skane University Hospital, S-20502 Malmö, Sweden

Email: [elin.dybjer@med.lu.se](mailto:elin.dybjer@med.lu.se)

**Peter M. Nilsson**

Department of Clinical Sciences, Lund University, Clinical Research Centre,

Skane University Hospital, S-20502 Malmö, Sweden

Email: peter.nilsson@med.lu.se

**Gunnar Engström**

Department of Clinical Sciences, Lund University, Clinical Research Centre,

Skane University Hospital, S-20502 Malmö, Sweden

Email: gunnar.engstrom@med.lu.se

**Catherine Helmer**

Univ. Bordeaux, Inserm, Bordeaux Population Health Research Center, team LEHA, UMR 1219, F-33000 Bordeaux, France

Email: Catherine.Helmer@u-bordeaux.fr

**Katarina Nägga**

Department of Acute Internal Medicine and Geriatrics, Linköping University, S-581 85 Linköping, Sweden. Clinical Memory Research Unit, Department of Clinical Sciences Malmö, Lund University, S-205 02 Malmö, Sweden

Email: katarina.nagga@med.lu.se

**Table S1. General linear models of adjusted mean cognitive test results across glucometabolic categories: *Post-hoc analyses* equivalent of Table 2, but dividing participants with diabetes into *short-term* diabetes (n=476) and *long-term* diabetes (n=77).**

|  | Model 1, n=2994 | Model 2, n=2994 |
| --- | --- | --- |
| A. MMSE  (points/100, normalized) |  |  |
| NGT | 80.4 (79.7-81.1) | 80.0 (79.3-80.8) |
| Pre-diabetes | 78.5 (77.6-79.4)** | 78.3 (77.4-79.2)** |
| Short-term diabetes | 79.0 (77.7-80.2)* | 80.0 (78.5-81.4) |
| Long-term diabetes | 74.7 (71.6-77.8)** | 76.9 (73.3-80.5) |
| P for trend across categories | **<0.001** | 0.078 |
| B. AQT  (time in seconds) |  |  |
| NGT | 130.8 (129.5-132.2) | 131.8 (130.3-133.1) |
| Pre-diabetes | 133.8 (132.0-135.5)* | 134.0 (132.2-135.8) |
| Short-term diabetes | 134.2 (131.8-136.5)* | 132.4 (129.8-135.2) |
| Long-term diabetes | 148.7 (142.3-155.4)*** | 144.9 (137.7-152.5)** |
| P for trend across categories | **<0.001** | **0.025** |
| C. Memory  (MMSE question 1+4, points/13) |  |  |
| NGT | 12.1 (12.1-12.2) | 12.1 (12.0-12.2) |
| Pre-diabetes | 12.0 (11.9-12.1)* | 12.0 (11.9-12.1)** |
| Short-term diabetes | 12.1 (12.0-12.2) | 12.1 (12.0-12.2) |
| Long-term diabetes | 11.6 (11.3-11.8)*** | 11.6 (11.3-11.9)** |
| P for trend across categories | **0.001** | **0.023** |
| D. Processing speed  (AQT part 1-2, time in seconds) |  |  |
| NGT | 60.6 (60.0-61.2) | 61.0 (60.4-61.6) |
| Pre-diabetes | 61.9 (61.2-62.7)* | 62.1 (61.3-62.9)* |
| Short-term diabetes | 62.2 (61.1-63.2)* | 61.3 (60.0-62.5) |
| Long-term diabetes | 68.8 (66.0-71.8)*** | 66.7 (63.6-70.1)** |
| P for trend across categories | **<0.001** | **0.026** |
| E. Executive functioning  (AQT part 3, time in seconds) |  |  |
| NGT | 69.9 (69.1-70.7) | 70.3 (69.5-71.2) |
| Pre-diabetes | 71.4 (70.4-72.5) | 71.5 (70.1-72.6) |
| Short-term diabetes | 71.5 (70.1-73.0)* | 70.7 (69.1-72.5) |
| Long-term diabetes | 79.4 (75.4-83.5)*** | 77.7 (73.2-82.4)* |
| P for trend across categories | **<0.001** | **0.047** |

Values are re-calculated from logarithmic values, apart from results of the MMSE, in which a normalization transformation method was used. All values are expressed as adjusted means (95% CI). For each cognitive outcome measurement, p-values for linear trends across categories are presented. Significant p-values for trend are highlighted in **bold** text.

*Model 1:* Adjusted for age, sex, education, physical activity level, smoking habits and alcohol consumption.

*Model 2:* Adjusted for factors in *Model 1* and cardiovascular factors: Systolic blood pressure, heart rate, c-f PWV, waist circumference, total cholesterol levels and medications (anti-hypertensive, anti-diabetic and lipid-lowering treatment).

**Table S2. Multiple linear regression analyses of linear relationships between *fasting* and *2h-glucose,* respectively, and cognitive *sub-domains*.**

|  | Model 1 | | Model 2 | |
| --- | --- | --- | --- | --- |
|  | **B** | **p** | **B** | **p** |
| ALL PARTICIPANTS |  |  |  |  |
| Fasting glucose (n=2991) |  |  |  |  |
| Memory (MMSE question 1+4) | -0.023 | **0.019** | -0.014 | 0.246 |
| Processing speed (AQT part 1-2) | 0.083 | **<0.001** | 0.024 | 0.342 |
| Executive functioning (AQT part 3) | 0.089 | **<0.001** | 0.043 | 0.150 |
|  |  |  |  |  |
| 2h-glucose (n=2671) |  |  |  |  |
| Memory (MMSE question 1+4) | -0.012 | **0.023** | -0.013 | **0.021** |
| Processing speed (AQT part 1-2) | 0.026 | **0.022** | 0.017 | 0.155 |
| Executive functioning (AQT part 3) | 0.038 | **0.006** | 0.027 | 0.064 |
|  |  |  |  |  |
| ALL WITHOUT DIABETES |  |  |  |  |
| Fasting glucose (n=2484) |  |  |  |  |
| Memory (MMSE question 1+4) | -0.031 | 0.051 | -0.029 | 0.100 |
| Processing speed (AQT part 1-2) | 0.101 | **0.002** | 0.045 | 0.212 |
| Executive functioning (AQT part 3) | 0.094 | **0.018** | 0.024 | 0.584 |
|  |  |  |  |  |
| 2-h glucose (n=2433) |  |  |  |  |
| Memory (MMSE question 1+4) | -0.015 | **0.023** | -0.016 | **0.019** |
| Processing speed (AQT part 1-2) | 0.033 | **0.020** | 0.023 | 0.117 |
| Executive functioning (AQT part 3) | 0.049 | **0.004** | 0.037 | **0.035** |

B = unstandardized regression coefficient. Significant p-values are highlighted in **bold** text.

*Model 1:* Adjusted for age, sex, education, physical activity level, smoking habits and alcohol consumption.

*Model 2:* Adjusted for factors in *Model 1* and cardiovascular factors: Systolic blood pressure, heart rate, c-f PWV, waist circumference, total cholesterol levels and medications (anti-hypertensive, anti-diabetic and lipid-lowering treatment).

**Table S3. General linear models of adjusted mean cognitive test results for participants with and without diabetes, using different classification methods for diabetes. Adjusted for age, sex, education, physical activity level, smoking habits and alcohol consumption.**

|  | Mean cognitive test result if not diabetes, (95% CI) | Mean cognitive test result if diabetes,  (95% CI) | n (n classified as diabetes) | P |
| --- | --- | --- | --- | --- |
| A. MMSE total score  (points/100, normalized) |  |  |  |  |
| Classification methods at *baseline* |  |  |  |  |
| HbA1c (diabetes defined as 5.6%  Mono-S equivalent to 6.5 % DCCT) | 79.8 (79.2-79.7) | 77.0 (74.6-79.4) | 2742 (129) | **0.047** |
| Fasting glucose at baseline (diabetes defined as glucose ≥ 7.0 mmol/L) | 79.5 (79.0-80.0) | 74.5 (71.0-77.9) | 2804 (62) | **0.005** |
| Self-reported diagnosis at baseline | 79.5 (79.2-79.7) | 78.3 (73.0-83.5) | 2967 (27) | 0.763 |
| Self-reported diabetes medication at baseline | 79.7 (79.4-80.0) | 76.5 (72.5-80.4) | 2449 (47) | 0.147 |
|  |  |  |  |  |
| Classification methods at *follow-up* |  |  |  |  |
| Fasting glucose at follow-up (diabetes defined as glucose ≥ 7.0 mmol/L) | 79.6 (79.0-80.1) | 78.9 (77.5-80.3) | 2611 (380) | 0.417 |
| 2-h glucose at follow-up (diabetes defined as glucose ≥ 11.1 mmol/L) | 79.8 (79.3-80.3) | 79.8 (77.8-81.9) | 2500 (171) | 0.851 |
| Self-reported diagnosis at follow-up | 79.7 (79.2-80.3) | 77.2 (75.7-78.8) | 2690 (304) | **0.003** |
| Self-reported diabetes medication at follow-up | 79.7 (79.2-80.3) | 76.5 (74.7-78.2) | 2754 (240) | **0.001** |
|  |  |  |  |  |
| B. AQT total score (s) |  |  |  |  |
| Classification methods at *baseline* |  |  |  |  |
| HbA1c (diabetes defined as ≥ 5.6% Mono-S equivalent to 6.5 % DCCT) | 135.4 (134.3-136.6) | 153.4 (148.0-158.7) | 2742 (129) | **<0.001** |
| Fasting glucose at baseline (diabetes defined as glucose ≥ 7.0 mmol/l) | 135.7 (134.6-136.8) | 157.7 (150.0-165.3) | 2804 (62) | **<0.001** |
| Self-reported diagnosis at baseline | 135.7 (135.2-136.3) | 143.0 (131.4-154.5) | 2967 (27) | 0.202 |
| Self-reported diabetes medication at baseline | 135.2 (134.0-136.4) | 148.4 (139.6-157.3) | 2449 (47) | **0.005** |
|  |  |  |  |  |
| Classification methods at *follow-up* |  |  |  |  |
| Fasting glucose at follow-up (diabetes defined as glucose ≥ 7.0 mmol/L) | 135.0 (133.8-136.2) | 141.3 (138.2-144.4) | 2611 (380) | **<0.001** |
| 2-h glucose at follow-up (diabetes defined as glucose ≥ 11.1 mmol/L) | 134.6 (133.5-135.8) | 136.5 (132.1-140.9) | 2500 (171) | 0.230 |
| Self-reported diagnosis at follow-up | 135.0 (133.9-136.2) | 142.7 (139.3-146.2) | 2690 (304) | **<0.001** |
| Self-reported diabetes medication at follow-up | 135.1 (134.0-136.2) | 144.1 (140.2-148.0) | 2754 (240) | **<0.001** |

Significant p-values are highlighted in **bold** text.

**Table S4. General linear models of adjusted mean AQT results for each glucometabolic category stratified for age and physical activity, adjusted for age, sex, education, physical activity level, smoking habits and alcohol consumption*.***

|  | N | Mean AQT score (seconds; 95% CI) |
| --- | --- | --- |
| 1. Stratified for age 2. Age ≥72 years |  |  |
| NGT | 721 | 139.8 (137.4-142.3) |
| Pre-diabetes | 490 | 143.8 (140.9-146.7) |
| Short-term diabetes | 259 | 146.0 (141.9-150.1)* |
| Long-term diabetes | 46 | 168.0 (158.4-177.5)*** |
| 1. Age < 72 years |  |  |
| NGT | 734 | 126.0 (124.3-127.7) |
| Pre-diabetes | 348 | 128.5 (126.0-131.0) |
| Short-term diabetes | 185 | 127.0 (123.6-130.5) |
| Long-term diabetes | 24 | 137.7 (128.4-147.0)* |
|  |  |  |
| 1. Stratified for physical activity 2. Regular exercise or training |  |  |
| NGT | 296 | 132.6 (129.4-135.7) |
| Pre-diabetes | 112 | 135.3 (130.2-140.5) |
| Short-term diabetes | 57 | 125.6 (118.5-132.8) |
| Long-term diabetes | 9 | 130.1 (111.7-148.5) |
| 1. Moderate exercise |  |  |
| NGT | 1086 | 133.1 (131.2-134.8) |
| Pre-diabetes | 670 | 136.9 (134.6-139.1)* |
| Short-term diabetes | 345 | 138.4 (135.3-141.5)** |
| Long-term diabetes | 49 | 153.4 (145.5-161.3)*** |
| 1. Sedentary spare time |  |  |
| NGT | 73 | 141.1 (133.1-149.1) |
| Pre-diabetes | 65 | 140.8 (131.9-149.7) |
| Short-term diabetes | 42 | 151.8 (141.5-162.1) |
| Long-term diabetes | 12 | 191.6 (171.1-212.1)*** |

* p<0.05, **p<0.01, ***p<0.001 of difference in mean test results between NGT and each of the other glucometabolic categories.
